# The American Laryngological Association

**Published:** 1886-06

**Authors:** 


THE AMERICAN LARYNGOLOGICAL ASSOCIATION held its annual meeting in Philadelphia, Pennsylvania, on May 27th, 28th and 29th. Dr. Harrison Allen presided. The number and the character of the papers presented during the session gave evidence of the interest taken in that specialty, and of the progress made in it in the last few years.

Professor E. F. Ingals, of this city, was elected president for the next year.

